# The Influence of Disorder on Thermotropic Nematic Liquid Crystals Phase Behavior

**DOI:** 10.3390/ijms10093971

**Published:** 2009-09-10

**Authors:** Vlad Popa-Nita, Ivan Gerlič, Samo Kralj

**Affiliations:** 1 Faculty of Physics, University of Bucharest, P. O. Box MG-11, Bucharest 077125, Romania; 2 Laboratory Physics of Complex Systems, Faculty of Natural Sciences and Mathematics, University of Maribor, Koroška 160, 2000 Maribor, Slovenia and Jožef Stefan Institute, Jamova 39, 1000 Ljubljana, Slovenia; E-Mails:Ivan.gerlic@uni-mb.si (I.G.);samo.kralj@uni-mb.si (S.K.)

**Keywords:** liquid crystals, random field, phase transitions, disorder, nematic-impurities mixture

## Abstract

We review the theoretical research on the influence of disorder on structure and phase behavior of condensed matter system exhibiting continuous symmetry breaking focusing on liquid crystal phase transitions. We discuss the main properties of liquid crystals as adequate systems in which several open questions with respect to the impact of disorder on universal phase and structural behavior could be explored. Main advantages of liquid crystalline materials and different experimental realizations of random field-type disorder imposed on liquid crystal phases are described.

## 1. Introduction

Understanding of phase and structural behavior of various systems which are randomly perturbed by some static origin of disorder is of considerable interest for different branches of physics [1, 2, 3]. Presence of disorder is practically unavoidable in any system (*e.g.,* it is enforced via “impurities”, thermal fluctuations...) and could significantly modify its properties quantitatively or even qualitatively. Randomly perturbed configurations often display several universal features. Such systems are therefore related from the mathematical point of view, although they could be seemingly completely different physically. Of particular interest are phases and structures, which are in the absence of disorder reached via a continuous symmetry breaking (CSB) transition [4] and exhibit long range order (LRO). Such configurations are extremely susceptible to disorder due to the existence of a Goldstone mode in the broken symmetry phase. The mode’s amplitude tend to diverge in the long wavelength limit because the energy costs of such excitations are negligible.

The pioneering studies have been mostly carried out in various randomly perturbed magnetic materials [1]. In order to understand and to control mean observed features minimal models have been developed in which magnetic spins interact through the Heisenberg or Ising form of the coupling interaction. In the absence of disorder, such systems exhibit continuous symmetry breaking when crossing from the paramagnetic to a magnetically ordered phase. The quenched disorder was either introduced via random field (RF) or random anisotropy magnetic (RAM) field-type coupling term exhibiting one fold and two fold axis symmetry [5, 6], respectively. Let us list some results of universal validity (*i.e.,* the systems of interest exibit CSB) which such models yield.

It was shown that the 1st order phase transitions become gradual as disorder exceeds the critical value [7]. With respect to the 2nd order phase transitions we refer to the Harris criterion [8]. It claims that the random bond disorder can change critical properties of a transition if *α >* 0. Here *α* stands for the critical coefficient describing thermal behavior of the specific heat. On the other hand, the renormalization study of the random anisotropy magnetic system [5] showed that the fixed point corresponding to the non-random critical behavior is unstable even with respect to infinitesimally weak disorder strength. This instability indicates either smoothing of the transition or a transformation into the 1st order transition. The latter effect could arise due to the static disorder affecting the Goldstone (also called gauge) type continuum field [9] in a reminiscent way as the thermal fluctuations of this field trigger the so called Halperin-Lubensky-Ma effect [10, 11] (*i.e.,* they transform the 2nd order phase transition into the 1st order). Note that the static disorder is much more efficient in comparison with the thermal one because of its persistent influence. It was shown that the static random field fluctuations could behave like thermal fluctuations with the lower marginal dimensionality increased by two [12]. Regarding the structure of the disordered magnetic phase, some studies predict that the symmetry broken phase exhibits a domain type structure characterized [12] by a single characteristic length scale *ξ_d_*. In that case the disordered phase exhibits a short range order (SRO). One of the cornerstone of statistical mechanics of disorder, the so called Imry-Ma theorem [12], claims that below the spatial dimension *d* = 4 the ordered state is unstable to an arbitrary weak random field that couples linearly with the order parameter. The latter exhibits continuous symmetry breaking transition on entering the lower symmetry phase. Furthermore, the scaling law *ξ_d_ ∝ w*^2/(*d*–4)^ is predicted, where *w* measures the disorder strength. However, some studies using different approaches predict for random anisotropy models and a weak enough disorder strength the algebraic decay of correlations [13, 14], the hallmark of quasi long range order (QLRO). But latter studies reveal that even in this case a characteristic length is present marking the distance above which the correlations decay rather weakly [15].

In recent years it has been shown that randomly perturbed liquid crystal (LC) phases and structures [16] present an adequate system in which several universal mechanism in the realm of statistical mechanics of disorder could be studied. Liquid crystal (LC) phases [17, 18] are often referred to as a fourth state of matter. In the literature, however, one can find also plasma or quark-gluon plasma as candidates for the same ranking. But already such a candidature indicates the important role this state of matter plays in nature. Most LC phases represent an intermediate state between ordinary liquids and crystals. In most cases they flow like a liquid. On the other hand they possess some long-range order (orientational or translational, or both) usually characterizing crystalline solids. They are also typical representatives of soft-matter systems [17, 18], in which a relatively small amount of locally supplied energy can cause a response on a macroscopic scale.

LCs have several extraordinary (mechanical, electrical and optical) properties which make them indispensable in biological systems and in our daily life [18]. But aside these facts LC phases offer a convenient tool to study or test several fundamental laws of physics [4, 19, 20]. For example, studies on the coarsening dynamics following a sudden phase transition from ordinary liquid to orientationally order nematic phase can shed light on phenomena in the early universe [21]. The reasons behind this are as follows. There exists a variety of LC phases, where every phase can exhibit various structures [17, 22] depending on boundary conditions and/or direction and strength of external electric or magnetic fields. The resulting states display extremely rich diversity of physical phenomena. Furthermore, LCs are adequate for experimental observations [17, 22]. The samples can be prepared relatively easily because LCs have liquid properties and can be shaped by boundaries. In addition their structure can be strongly influenced by conditions at the confining surface [23] or by external electric or magnetic fields. The characteristic lengths and time responses to locally induced perturbations are accessible to conventional experiments. In particular, several optical measurements can be used because LCs are transparent and show an optical anisotropy strongly linked to electric and magnetic anisotropy.

In order to study influence of random field-type disorder on phase and structural behavior one commonly enforces disorder to LCs either by mixing them with “impurities” or confining LCs to various porous matrices [16]. As impurities, aerosil nanoparticles [3, 24, 25, 26] are typically used, which can form qualitatively different random networks as the concentration of aerosils is varied. In terms of porous confinements, aerogels [27, 28], Russian glasses [29], Vycor glasses [30], and Controlled Pore Glasses (CPG) [31, 32] are mostly used. Therefore, by choosing different LCs phases and perturbing porous matrices or “impurities” one can impose different types and strength of disorder.

The paper is organized as follows. In Section II, we present a brief overview on liquid crystals, containing the principal characteristics and properties of the three main classes and the definition of the nematic orientational order parameter. In Section III, the basic properties of principal models of nematic-isotropic phase transition are shown. In Section IV, the influence of the random field on structural and phase transition properties is theoretically described in the framework of a mesoscopic phenomenological model. The experimental observations which support the theoretical model are also described. In the final Section V, we draw some conclusions.

## 2. Liquid Crystals: A Brief Overview

Liquid crystals were discovered in 1861 by Planer, confirmed in 1888 by Reinitzer [33], named by Otto Lehmann in 1889 [34] and understood in terms of the local ordering of elongated particles and classified by Georges Friedel in 1922 [35].

Liquid crystals or mesophases constitute a state of matter whose physical properties are intermediate between those of an isotropic liquid and a crystalline solid [36, 37, 38, 39, 40, 41, 42, 43, 44, 45]. The existing long-range order in a crystal consisting of anisotropic molecules is both *positional* (the molecules occupy specific sites in a lattice) and *orientational* (the molecular axes point in specific directions). When such a crystal is heated from the solid phase it is possible that both types of order (positional and orientational) disappear at the same temperature and the resulting phase is an isotropic liquid. Alternatively, it is possible that one type of ordering survives until a higher transition temperature. If the orientational order disappears leaving the positional order intact the corresponding phase is called plastic crystal. On the contrary, if the positional order either fully or partially disappears while some degree of orientational order is maintained, the resulted phase is called liquid crystal.

Liquid crystals possess many of the mechanical properties of a liquid, *e.g.,* high fluidity and the inability to support shear, but on the other hand, they have some properties similar to crystals, *e.g.,* they are birefringent and have anisotropic magnetic and electric susceptibilities. The relative large value of latent heat (∝ 250 J/g) at crystal-liquid crystal phase transition indicates that most of the order of a crystal is lost when it transform to a mesophase. On the contrary, the latent heat at liquid crystal-isotropic phase transition is much smaller, typically about 5 J/g.

The constituent organic molecules of liquid crystalline materials could be in general very different, but it is essential that they are anisotropic and rigid for at least some portion of the molecule length (since it must maintain an elongated shape in order to produce interactions that favor alignment). The liquid crystals composed from rod-like molecules are called “calamitics”, while those formed by disk-shape molecules are known as “discotic” [41, 42].

Two types of liquid crystals must be differentiated: (i) thermotropic and (ii) lyotropic. The transitions involving thermotropic liquid crystals are effected by changing temperature. Lyotropic liquid crystals are found in solutions and concentration is the important controllable parameter. Usually a lyotropic liquid crystal molecule combines a hydrophobic group at one end with a hydrophilic group at the other end. These amphiphilic molecules forms ordered structures (micelles, vesicle, lamellar phases or reversed phases) in both polar and non-polar solvents as in the case of soaps and various phospholipids [45].

Another important class of liquid crystals is derived from certain macromolecules, usually in solution but sometimes even in the pure state. These liquid crystals are known as “liquid crystal polymers” [43, 46, 47, 48]. They can be divided corresponding either to molecular anisotropy or location of the mesogenic group. On the basis of molecular anisotropy two types of structures are possible. In the first type the mesomorphism is due to rigid macromolecule as a whole or from several units made up of rigid chain segments, whereas in the second class only the monomer unit possesses a mesogenic structure connected to the rest of molecule via a flexible spacer. On the basis of location of the mesogenic unit the liquid crystal polymers can be divided into: main-chain liquid crystal polymers and side-chain liquid crystal polymers [44].

Based upon their symmetry, Friedel distinguishes three major classes of liquid crystals—nematics, cholesterics, and smectics.

### 2.1. Nematic, Cholesteric and Smectic Liquid Crystals

**Figure 1.**
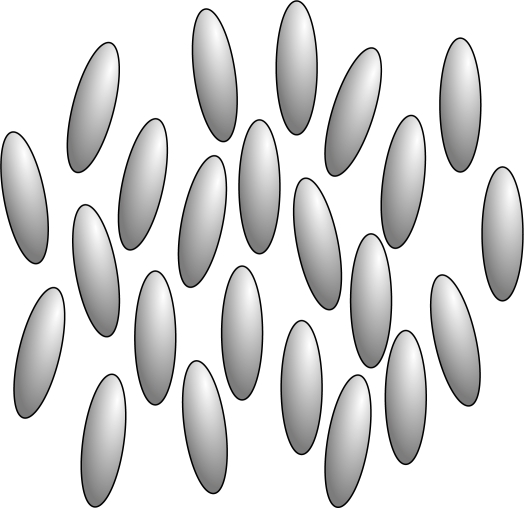
The arrangement of molecules in the nematic phase.

An isotropic liquid possesses full translational and orientational symmetry *T*(3) *× O*(3). At the transition in the nematic phase the translational symmetry *T*(3) remains the same, but the rotational symmetry *O*(3) is broken. In the simplest uniaxial nematic structure the group *O*(3) is replaced by one of the uniaxial symmetry groups *D_∞_* or *D_∞ h_*. The nematic molecular order is shown schematically in Figure 1.

In this phase there exists no long-range positional order (between the centers of mass of the molecules). The molecules tend to be parallel to some common axis, labeled by a unit vector $\vec{n}$, called “director”. The direction of $\vec{n}$ is arbitrary in space; in practice it is imposed by external forces such as the guiding effect of the wall of the container or electric and magnetic fields. The states of director $\vec{n}$ and $-\vec{n}$ are indistinguishable even if the molecules may be polar (no ferroelectricity has been observed). Microscopically, an equal number of molecules points “up” and “down”. The uniaxial nematic phase is characterized by rotational symmetry of the system around the director $\vec{n}$. One important consequence of this is that there are two different principal components of the second rank tensorial properties as, *e.g.,* magnetic susceptibility, dielectric constant, or refractive indices. In general two uniaxial nematic phases can be distinguished: (i) prolate nematic phase which occur for rodlike molecules and (ii) oblate nematic phase characteristic for disklike molecules.

The biaxial nematic phase results due to further breaking of the rotational symmetry and is characterized by three orthogonal directors, the Goldstone modes $\vec{n}$, $\vec{l}$, and $\vec{m}=\vec{n}\times \vec{l}$. Due to the lack of polarity of the known biaxial nematics, the directions $\vec{n}=-\vec{n}$, $\vec{m}=-\vec{m}$, and $\vec{l}=-\vec{l}$ are equivalent. The biaxial nematic phase possess a *D*_2 *h*_ point group symmetry and the corresponding second rank tensor properties have three different principal components.

**Figure 2.**
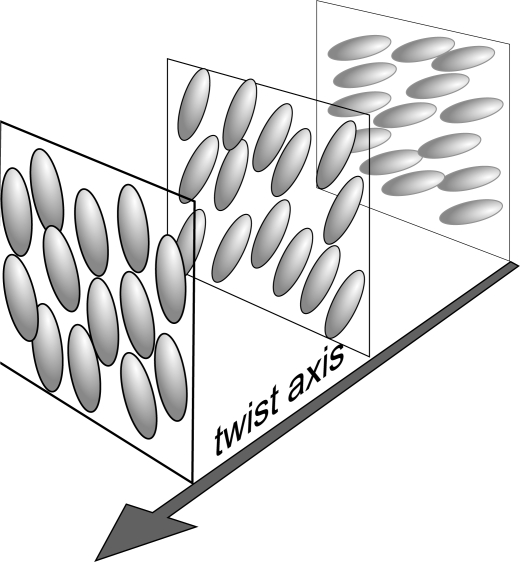
The arrangement of molecules in the cholesteric phase; the plans have been drawn for convenience, but do not have any specific physical meaning.

If the liquid crystal molecules are chiral (lacking inversion symmetry), the uniaxial nematic phase is replaced by the chiral nematic phase or twisted nematic phase or cholesteric phase. Locally, a cholesteric is very similar to a nematic. Again, the centers of mass have no long-range order and the molecular orientation shows a preferred axis labeled by a director $\vec{n}$. However, $\vec{n}$ is not constant in space. The preferred conformation is helical (see Figure 2). The helical distortion can be found either in pure cholesterol esters (which are chiral) or by dissolving in a nematic a fluid composed by chiral molecules.

When the crystalline order is lost in two dimensions, one obtains the two-dimensional liquids, called smectics. Smectics liquid crystals have layered structures with a well-defined interlayer spacing that can be measured by X-ray diffraction. In addition to the orientational order, the smectic molecules exhibit some correlations in their position. In most smectics the molecules are mobile in two directions and can rotate around one axis. The interlayer attractions are weak compared with the lateral forces between molecules and the layers are able to slide over one another relatively easily which gives rise to fluid property of the system with higher viscosity than nematics. When smectic and nematic phases are found in one compound, the nematic phase is almost always found at higher temperatures with the exception of reentrant nematic phase.

**Figure 3.**
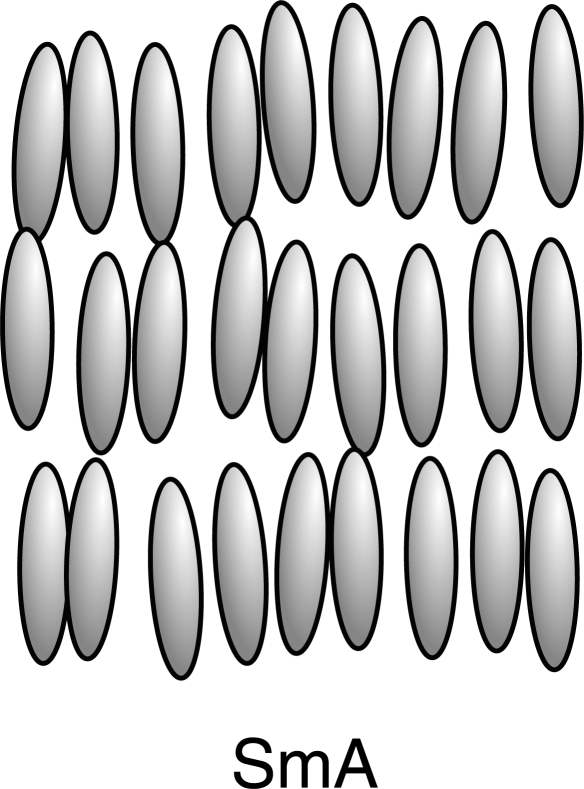
The arrangement of molecules in the smectic A phase.

The observed smectic phases differ from each other in the way of layer formation and the existing order inside the layer [42, 44, 45]. The simplest is the smectic A phase (the phase recognized by G. Friedel (see Figure 3) with symmetry *T*(2) *× D_∞ h_*. In the phase smectic A, the average molecular axis is normal to the smectics layer. The layer thickness can vary from a value close to the length of the molecule in thermotropic liquid crystals to the periodicity as large as several *μm* in lyotropics. Inside each layer, the centers of mass of molecules show no long-range order and the molecules have considerably freedom of rotation around their long axis; each layer is a two-dimensional fluid and the whole structure may be defined as an orientational ordered fluid on which is superimposed a one-dimensional density wave. The distortions due to flexibility of layers leads to optical patterns known as focal conic textures [45]. When temperature is decreased, the smectic A phase may transform into a phase possessing a lower symmetry. The breaking of *D_∞ h_* may lead to the appearance of tilting of molecules relative to the smectic layers. This phase is called smectic C which possesses *T*(2) *× C*_2 *h*_ symmetry.

**Figure 4.**
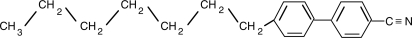
The chemical formula of octyl-4-cyanobiphenyl (8CB).

As an example of compound that can be in both nematic and smectic A phases we cite the very much used octyl-4-cyanobiphenyl (8CB) with chemical formula shown in Figure 4, which is smectic A between 24 °C and 34 °C, and nematic between 34 °C and 42.6 °C.

### 2.2. Order Parameters

The fundamental characteristics of a liquid crystal is the presence of long-range orientational order while the positional order is either absent (nematic phase) or limited (smectic phases). One phase differs from another by its symmetry. The transition between phases of different symmetry can be described in terms of an order parameter *Q* that must satisfy the following requirements: (i) *Q* = 0 in the more symmetric (less ordered) phase and (ii) *Q ≠* 0 in the less symmetric (more ordered) phase. These requirements do not define the order parameter in a unique way, but in spite of this arbitrariness, in many cases the order parameter can be defined in a natural way. In the case of liquid-vapor transition, the order parameter is a scalar and is defined as the difference in density between the two phases. In the case of ferromagnetic transitions, the order parameter is the magnetization, a vector with three components. In general the order parameter can be defined as follows. Identify the symmetry of a phase. Next take the basis functions of the irreducible representations of this group symmetry. Expand the density distribution in the basis functions. The coefficients of this expansion are the order parameters. The order parameter as a quantitative measure of order in the system is used in such a diverse phase transitions as solid-solid, liquid-solid, in liquid crystals, superconductivity, superfluid helium, ferromagnetic-paramagnetic and polymeric systems.

#### Nematic order parameter

The macroscopic orientational nematic order parameter is a traceless, symmetric, second rank tensor with components given by [42, 49]

(1) $$Q_{\alpha \beta }=\frac{1}{2}\;S\;(3n_{\alpha }n_{\beta }-\delta_{\alpha \beta })+\frac{\sqrt{3}}{2}\;\eta \;(l_{\alpha }l_{\beta }-m_{\alpha }m_{\beta })$$

where $\vec{m}=\vec{n}\times \vec{l}$. The eigenvalue of **Q** largest in absolute magnitude gives the degree of orientation in the preferred direction, the corresponding eigenvector identifying the preferred direction $\vec{n}$ (the director in the uniaxial nematic phase). The difference between the remaining two eigenvalues characterizes the degree of biaxiality with the biaxiality axis $\vec{l}$ specified by the eigenvector corresponding to the second largest eigenvalue. The general biaxial state is thus specified by five parameters, which may be considered either as (i) the five independent elements of **Q**, or as (ii) the strength of uniaxial ordering *S*, the biaxial scalar amplitude *η*, two angles to specify the orientation of the director $\vec{n}$, and a third angle to specify the orientation of $\vec{l}$ about $\vec{n}$. Because **Q** is symmetric, it is always possible to find a reference frame in which it is diagonal, with its eigenvalues as diagonal elements:

(2) $$Q=\left(\begin{array}{ccc} \frac{1}{2}(-S+\sqrt{3}\eta ) & 0 & 0\\ 0 & \frac{1}{2}(-S-\sqrt{3}\eta ) & 0\\ 0 & 0 & S \end{array}\right)$$

A uniaxial nematic state is described by the condition that two eigenvalues of **Q** coincide, while a biaxial nematic state is characterized by the condition that all eigenvalues of **Q** be distinct.

The microscopic ordered parameters *S* and *η* are related to specific molecular models and are obtained as expansion coefficients of the singlet orientational distribution function in a suitable base set [42, 44, 50].

The uniaxial nematic state state is specified by only three parameters: the magnitude *S* and two angles to orient the director. To define the microscopic orientational order parameters of the nematic, we start with the singlet orientational distribution function, defined as

(3) $$f\;(\Omega )=\frac{V}{Z_{N}}\;\int \text{exp}\;\left[-\beta U\;(X_{1},\;X_{2},\;\ldots X_{N})\right]dX_{1}dX_{2}\ldots dX_{N}$$

which is normalized to unity

(4) ∫f(Ω) dΩ=1

*U* (*X*_1_*, X*_2_, ...*X_N_*) is the potential energy of *N* particles and *β* = 1/*k_B_T* with *k_B_* the Boltzmann constant. *Z_N_* is the configurational partition function of the system

(5) $$Z_{N}=\int \exp\;[-\beta U\;(X_{1},\;X_{2},\ldots X_{N})]dX_{1}dX_{2}\ldots dX_{N}$$

Here the $X_{i}=({\vec{r}}_{i},\Omega_{i})$ specify the position ${\vec{r}}_{i}$ of the center of mass of the ith molecule and its orientation Ω*_i_* described by the two angles (θ*_i_*, φ*_i_*).

If the mesogenic molecules possess cylindrical symmetry, *f*(Ω) depends only on the angle *θ* between the director and the molecular symmetry axis and the orientational order parameters are defined by

(6) $$〈P_{l}〉=\int P_{l}(\text{cos}\;\theta )f(\theta )d\Omega$$

where *P_l_* are the even Legendre polynomials. From knowledge of *f* (θ) all the orientational order parameters 〈*P*_2_〉, *〈P*_4_〉, etc., can be calculated. The most important one is usually called the scalar order parameter *S* defined as $S=〈P_{2}〉=\frac{1}{2}(3〈{\text{cos}}^{2}\theta 〉-1)$. In the isotropic liquid where all orientations are equally probable, *S* = 0. In a fully oriented nematic phase where all the symmetry axes of the molecules are parallel with the director *S* = 1, and in fully oriented nematic phase where the symmetry axes of the molecules are distributed in the plane orthogonal to the director, *S* = −1/2.

## 3. Nematic-Isotropic Phase Transition: Theoretical Models

The experimental observations [41, 43] using various techniques [38, 39] show that the scalar order parameter *S* decreases monotonically as the temperature is raised in the nematic range and drops abruptly to zero at the nematic-isotropic phase transition temperature (*T_NI_*). In the case of uniaxial nematic the jump of *S* at nematic-isotropic phase transition has a value in the range of 0.25 − 0.5 depending on the mesogenic material and usually the latent heat is only 1 kJ/mol. Thus, the nematic-isotropic phase transition is *weakly* (small latent heat) *first order* (the jump of the order parameter). This leads to large pretransitional [39, 41] abrupt increases of response functions as specific heat, thermal expansion and isothermal compressibility near *T_NI_*. The changes of entropy and density associated with this transition are typically only a few percent of the corresponding values for solid-nematic phase transition. The theories of nematic phase and of nematic-isotropic phase transition have been developed in two directions: (i) microscopic and (ii) mesoscopic.

### 3.1. Microscopic Models

In the microscopic models we deal with the system at the molecular level and we start the calculations on the basis of partition function or the exact density functionals of the free energy. Accordingly, for a complete and satisfactory microscopic theory a knowledge of the intermolecular interaction is necessary. Unfortunately such a knowledge is almost entirely lacking. Even if the essentials of the intermolecular interaction are known, the successful application of the theory is very difficult due to enormous calculation problems. Owing to these difficulties the model potentials have been introduced, *i.e.,* the most relevant characteristics of the molecules and their interactions are represented in term of simple models. The purpose of the remaining of this section is not the detailed description of all the technical details of the microscopic models, but rather the presentation of their basic properties.

#### The Onsager Theory

The first extremely successful molecular model (of mean field type) of liquid crystal ordering was developed by Onsager [51] in order to describe the ordering in the Tobacco Mosaic Virus (TMV) solution. TMV are rigid cylindrically molecules of length *L* = 300 nm and diameter *D* = 18 nm, each composed of 2130 proteins arranged in a helical way and formed a hollow cylinder. The diameter of the cylinder hole in the cylinder is about 2 nm. TMV in water solution forms a birefringent liquid crystal phase when its concentration exceeds 2%. In the model the molecules are rigid rods of length *L* and diameter *D*, which interact only via the hard-core repulsive forces. The rigid rod is usually modeled by a spherocylinder, which is a straight circular cylinder capped on each end by a hemisphere of the same radius. Since in the system the attractive interactions are absent, the temperature sets only the energy scale. The calculation of Onsager is based on a cluster expansion for the free energy as a functional of the singlet orientational distribution function and truncating the series after the second virial coefficient [44, 51, 52].

The configurational free energy (per rod) of a system of hard rods can be expressed as

(7) $$F=F_{0}+k_{B}T\;\left(\int f(\Omega )\;\text{ln}\;[4\pi \;f\;(\Omega )c]d\Omega +\frac{1}{2}c\;\int \int f(\Omega_{1})\;f\;(\Omega_{2})\;V_{\text{exc}}(\Omega_{12})\;d\Omega_{1}d\Omega_{2}\right)$$

where *F*_0_ is an additive constant and *c* is the concentration of rods. The second term in Equation (7) describes the drop in entropy associated with molecular alignment, while the third term describes the excluded volume effect; *V_exc_*(Ω*_i_*, Ω*_j_*) = *V_exc_*(Ω*_ij_*) is the mutual excluded volume of two rods with orientations Ω*_i_* and Ω*j*.

The singlet orientational distribution function can be determined by minimizing the free energy subject to the constraint ∫ *f*(Ω)*d*Ω = 1. The result is the self-consistent equation

(8) $$\text{ln}[4\pi \;f\;(\Omega_{1})]=\lambda -1-c\int V_{\text{exc}}(\Omega_{12})\;f\;(\Omega_{2})d\Omega_{2}$$

where *λ* is the Lagrange multiplier determined by the normalized condition.

Onsager obtained an approximate variational solution which is based on a trial function of the form *f* = (const) cosh(*α* cos *θ*), where *α* is a variational parameter and *θ* is the angle between the molecular axis and the nematic axis. It was found [51] that the system exhibits an abrupt first-order phase transition from the isotropic to the nematic phase characterized by the following values of volume fractions of the isotropic *φ*_iso_, nematic phase *φ*_nem_, and order parameter *S* at coexistence

(9) $$\varphi_{\text{iso}}=3.3D/L,\;\;\;\;\;\varphi_{\text{nem}}=4.5D/L,\;\;\;\;S_{N\;I}=0.84$$

The Onsager model of nematic-isotropic phase transition is exact in the limit of the infinite length to width ratio *L/D* and is was argued that the truncation of the cluster expansion series after the second virial coefficient can be quantitatively justified only for very long rods *L/D >* 100, which are typical of polymeric system. For the shorter rods, the Onsager theory tends to become inaccurate at the high densities typical of nematic-isotropic transition, and more refined approaches have been developed [44].

#### The Maier–Saupe Model

The mean-field model of Maier and Saupe [42, 44] assumed that the nematic orientational order is caused by the anisotropic part of the dispersion interaction between molecules. Now, it is well accepted that the exact nature of the interaction need not be specified for the development of the theory. To obtain the results of the Maier Saupe theory, only an anisotropic potential with a particular dependence on the molecular orientations is required [53].

The standard Maier–Saupe free energy difference between nematic and isotropic phases can be written as

(10) $$\Delta F(S)=-TS(S)-\frac{1}{2}U\;S^{2}$$

where S(S) is the decrease in entropy due to the alignment of the molecules and the last term represents the anisotropic contribution to the internal energy. We now recall briefly the mean field calculation of *S*(*T*).

The trial singlet orientational distribution function is chosen to be given by

(11) $$f(\Omega )={\left(4\pi Z\right)}^{-1}\exp[mP_{2}(\text{cos}\;\theta )]$$

with

(12) $$Z(m)={\left(4\pi \right)}^{-1}\int \exp[mP_{2}(\text{cos}\;\theta )]d\Omega$$

where *m* is a dimensionless measure of the mean field aligning a molecule and *P*_2_(cos *θ*) = (3 cos^2^ *θ −*1)/2 is the second order Legendre polynomial. The conjugated nematic order parameter *S*(*m*) is related to *Z* by

(13) $$S(m)=\int P_{2}(\text{cos}\theta )f(\Omega )d\Omega =\frac{1}{Z}\frac{dZ}{dm}$$

The entropy change associated with the nematic orientational order is given by

(14) $$\Delta S=-k_{B}\int f(\Omega )\text{ln}[4\pi \;f(\Omega )]d\Omega$$

The order parameter is calculated by minimizing Δ*F* with respect to *S* which leads to

(15) $$S(m)=\frac{k_{B}T}{U}m$$

where *S*(*m*) is calculated from Equation (13). The results lead to a first order nematic-isotropic (NI) phase transition at

(16) $$\frac{U}{k_{B}T_{N\;I}}=4.541$$

and

(17) $$S_{N\;I}=0.429$$

The possible extensions of the Maier–Saupe theory have been presented *in extenso* in [44].

#### The van der Waals (vdW) and density functional theories(DFT)

A complete molecular theory of the mesophases must include both anisotropic short range repulsive and long-range attractive forces. The vdW types theories [44, 54, 55] recognize that the predominant factor in determining the liquid crystalline stability are the short range repulsive forces and that the role of the attractive long range forces is, to a first approximation, merely to provide a negative, spatially uniform mean field in which the molecules move. All the vdW type theories can be derived from a perturbation method, within the mean field approximation, considering only the first order approximation term. Despite its quantitative inadequacies, the vdW approach indicates that the anisotropy of the short range intermolecular repulsions plays a major role in determining nematic orientational order and stability and cannot be neglected, even to a first approximation.

The classical version and development in the present form have been discussed at length in other reviews [44, 56, 57, 58]. The main idea of the theory is that in the presence of a potential, the density is in fact *uniquely* determined. The net result is that the grand thermodynamic potential can be written as a functional of the density. Minimizing the grand thermodynamic potential, the thermodynamic properties of the system are obtained.

### 3.2. The Phenomenological Landau–de Gennes Theory

The Landau–de Gennes theory of phase transitions in liquid crystals [36, 37, 42, 59] is a phenomenological mesoscopic theory which apply the general rules of Landau theory [60] originally proposed by Landau for second order phase transitions in crystals. The basic idea is that sufficiently closed to the order-disorder phase transition the thermodynamic potential can be expanded in a series of powers of the order parameter. The motivation is derived from the continuity of the order parameter at the second order phase transition. Hence the Landau original procedure is, in principle, restricted to second order phase transition. In this section the application of Landau–de Gennes theory to nematic-isotropic phase transition will be summarized.

The most general form of the free energy *F* of the system is given by

(18) $$F=\int (f_{h}+f_{e})dV+\int f_{s}dA$$

where *V* is the nematic volume and *A* is the area of the interface confining it.

Near the nematic-isotropic transition point, in a mesoscopic approach the phenomenological Landau–de Gennes form of the homogeneous free energy density *f_h_* term is given by

(19) $$f_{h}(T,Q)=f(T,0)+a(T-T*)\;\text{tr}\;{\text{Q}}^{2}-B\;\text{tr}\;{\text{Q}}^{3}+C{\left(\text{tr}\;{\text{Q}}^{2}\right)}^{2}$$

where *f*(*T,* 0) is the free energy density of the isotropic phase (usually the free energy is normalized such as *f*(*T,* 0) = 0), T* is the undercooling limit temperature of the isotropic phase and the coefficients *a*, *B*, and *C* depend only on the substance (for 8CB they have the following values: *a* = 3.3 *·* 10^4^ J/K cm^3^, *B* = 8.9 · 10^5^ J/cm^3^, and *C* = 5.6 · 10^5^ J/cm^3^ [45]). They do not have a physical significance and their values are obtained by fitting the theoretical values of some quantities (i. e., the values of the order parameter at the transition, of *T_NI_* − T*, and of latent heat, respectively) with experimental data. The main properties of the expansion (19) can be summarized as follows:

- There is no term linear in *Q*. This allows for the possibility of an isotropic phase (*Q* = 0). In the case of external fields, a linear term has to be included and the isotropic phase transforms into a paranematic phase (a phase with a very low degree of orientational order).
- If *B >* 0, the transition is first order (the order parameter changes discontinuous at the transition), while if *B* = 0 the transition is second order (the order parameter is continuous at the transition, but its first derivative with respect to temperature is discontinuous). The other possible mechanism to mimic the first order phase transition is to consider *B* = 0 and *C <* 0. In this case a stabilizing six order term with *E >* 0 is required.
- To ensure stability of the nematic phase, *C* must be positive.

Using the representation (2) for the order parameter, the corresponding homogeneous Landau–de Gennes free energy density (19) is given by:

(20) $$f_{h}(T,S,\eta )=\frac{3}{2}a(T-T*)(S^{2}+\eta^{2})-\frac{3}{4}BS(S^{2}-3\eta^{2})+\frac{9}{4}C{\left(S^{2}+\eta^{2}\right)}^{2}$$

The uniaxial nematic state (with the choice of $\vec{n}$ as the director, i. e., the vector with the maximum eigenvalue of **Q**) corresponds to *η* = 0, value obtained minimizing *f*(*T, S, η*) with respect to *η*. The corresponding order parameter is defined as (see Equation (1))

(21) $$Q_{a\beta }=\frac{1}{2}S(3n_{\alpha }n_{\beta }-\delta_{a\beta })$$

while the corresponding homogeneous free energy density is given by

(22) $$f_{h}(T,S)=\frac{3}{2}a(T-T*)S^{2}-\frac{3}{4}BS^{3}+\frac{9}{4}CS^{4}$$

which describes a weakly first order uniaxial nematic-isotropic phase transition. At nematic-isotropic phase transition temperature *T* = *T_NI_* = T* + *B*^2^/24*aC* the two phases, uniaxial nematic (*S*_nem_ = *B/*6*C*) and isotropic (*S*_iso_ = 0) coexist in equilibrium. The minimum corresponding to the nematic phase becomes metastable for *T_NI_ < T < T*^+^ = T* + 3*B*^2^/64*aC* and does not exist for *T > T*^+^, the superheated limit temperature of the nematic phase. The minimum corresponding to the isotropic phase is metastable for T* < T < T_NI_ and does not occur when T < T*, the supercooling limit of the isotropic phase. The latent heat at the transition is given by Δ*H_NI_* = *aB*^2^*T_NI_*/24*C*^2^.

The elastic free energy density (*f_e_*) opposes the spatial variation in **Q** and is expressed as [42]:

(23) $$f_{e}=LQ_{\alpha \beta ,\gamma }Q_{\alpha \beta ,\gamma }$$

where *L* is a representative bare nematic elastic constant. In the Frank description of the nematic ordering, this form of *f_e_* corresponds to the approximation of equal Frank elastic constants.

The most essential surface contribution (anchoring term) of the free energy is given by

(24) $$f_{s}=-\frac{W}{3}{\vec{e}}_{s}\cdot \text{Q}\cdot {\vec{e}}_{s}$$

where *W* is the anchoring energy. The anchoring term tends to align liquid crystal molecules along the unit vector ${\vec{e}}_{s}$, referred to as the easy axis.

For the uniaxial ordering, using the representation (21) for the order parameter, the homogeneous free energy density term is given by Equation (22), while the other two terms can be expressed as

(25) $$f_{e}=\frac{3}{2}L|\nabla S{|}^{2}+\frac{9}{4}LS^{2}|\nabla \vec{n}{|}^{2}$$

(26) $$f_{s}=-\frac{W}{2}S\left({\left(\vec{n}\cdot {\vec{e}}_{s}\right)}^{2}-\frac{1}{3}\right)$$

### 3.3. Relation between Landau–de Gennes and Maier–Saupe Free Energies for Uniaxial Nematics

To obtain an insight into the structure and origin of the phenomenological parameters *a*, *B*, and *C* of the homogeneous Landau–de Gennes free energy, we start from the mean field Maier–Saupe approach on the molecular level. For uniaxial nematic composed of cylindrically symmetric molecules, the states with orientational order are distinguished by an axis of symmetry, the director $\vec{n}$ and an infinite set of scalar order parameters

(27) $${\bar{P}}_{2n}=\int P_{2n}(\text{cos}\;\theta )\;f(\Omega )d\Omega$$

where *n >* 0 is an integer, *P*_2*n*_(cos *θ*) are the Legendre polynomials and *f* (Ω) is the singlet orientational distribution function defined in Equation (3). Among these order parameters, *P̅*_2_ is by far the most important both from theoretical and experimental point of view, and is referred to as the nematic scalar order parameter *S*. In the isotropic phase all orientations are equivalent and *f*(Ω) = 1/4*π*; *S* = 0. Expanding the singlet orientational distribution function in terms of Legendre polynomials and keeping only the leading term, we obtain

(28) $$f(\Omega )=\frac{1}{4\pi }\sum_{n=0}^{\infty }\frac{{\bar{P}}_{2n}}{4n+1}P_{2n}(\text{cos}\;\theta )\approx \frac{1}{4\pi }\left(1+\frac{1}{5}SP_{2}(\text{cos}\;\theta )\right)$$

Using Equation (14), Equation (10) can now be written as a power series in *S* [61, 62]

(29) $$\Delta f_{h}(S)=\frac{k_{B}}{250}(T-T*)S^{2}-\frac{k_{B}T}{13125}S^{3}+\frac{k_{B}T}{87500}S^{4}+\ldots$$

where T* = 125*U/k_B_*. In the spirit of Landau–type approach, the dependence on temperature in cubic and quadratic terms in Equation (29) can be neglected and the free energy density of the nematic uniaxial phase takes the well-known Landau–de Gennes expression (22). It is to be noted that the T* term in Landau-de Gennes expansion is the only term that originates from the interaction Maier–Saupe term, the other terms originate from the Maier–Saupe entropy.

## 4. Influence of Random Field on Structural and Phase Transition Properties: A Mesoscopic Phenomenological Model

Liquid crystals, by virtue of their fluidity, intrinsically soft elasticity and experimental accessibility, offer opportunities for the study of the structural and dynamical effects of quenched disorder, which can be introduced, for example, by confinement within appropriate random porous media [16]. Such studies are also of interest in connection with composite electro-optic materials composed of mixtures of liquid crystal and impurities, or colloids, or nanoparticles. In such mixtures, a degree of randomness is imposed on the liquid crystal by the second component. The changes of the physical behavior of liquid crystal in such systems are generated by three main causes: (i) surface effects, (ii) confinement, and (iii) randomness. In this section we focus only on the theoretical study of the influence of randomness on phase transitions properties in liquid crystals.

Numerous studies [3, 9, 63, 64, 65, 66, 67, 68, 69, 70, 71, 72, 73] have suggested that similar random field type models may provide a useful context within which to understand the influence of randomness on physical properties of liquid crystals. These models assume that the porous medium (or the second component in a mixture) imposes a local field which has a randomizing effect on the nematic director. Such a randomizing field depending neither on temperature nor on the phase structure of liquid crystal, is generally refereed to as *quenched disorder*. The new equilibrium structure of the liquid crystal is the consequence of the competition between the quenched disorder provided by the randomness and the elastic force which tends to minimize the distortions of the nematic director.

In the following we analyze structural and phase behavior of a nematic liquid crystal experiencing quenched random anisotropy field (RAN) disorder in the simplest possible mean-field type approach. We first consider a typical domain structure in the nematic director field. We show that a domain structure is practically always expected, at least temporarily, if a lower symmetry (*i.e.,* the nematic) phase is reached via a continuous symmetry breaking phase transition. If disorder is present it could stabilize domains, the characteristic size of which is determined by the disorder strength. We show that such systems might display pronounced memory effects. Then we discuss phase behavior across the isotropic-nematic phase transition assuming the domain-type nematic structure. We consider case of (i) nematic LC, and (ii) nematic-non nematogenic mixture experiencing RAN. Finally we present experimental systems in which described behavior is observed.

### 4.1. Structural Behavior : Domain-Type Patterns

The main part of the paper is concerned with phase and structural behavior of randomly perturbed systems in which domain-type patterns are assumed. For this purpose we list evidences supporting this description in phases or structures exhibiting continuous symmetry breaking, where the lower symmetry phase possesses long range order (or at least quasi long range order).

In general, at the mesoscopic continuum level the degree of ordering in the broken symmetry configuration is determined by an order parameter field (OPF). On the other hand a selected symmetry breaking state is described by a symmetry breaking field (SBF), also referred to as a gauge field [17]. In absence of external ordering fields OPF and SBF in general respond to local perturbations on apparently different length scales (*e.g.,* these sizes can be comparable in severely confined structures, where the geometrical size characterizing the confinement is comparable to the relevant order parameter correlation length). OPFs relax towards an equilibrium value on distances comparable to order parameter correlation length *ξ*, which reflects intrinsic material properties [17]. In a thermotropic LC phase values of *ξ* are typically comparable to several nm far bellow the phase transition temperature [17]. On the other hand SBF is susceptible to a large scale geometry of the sample [17, 74]. For example, if a frustration is imposed on SBF via conflicting boundary conditions at surfaces separated by a distance *R*, then the field exhibits gradual changes over all the available space. This is due to the to the existence of Goldstone fluctuation modes in SBFs.

An example of a continuous symmetry breaking in orientational ordering represents the isotropic to nematic liquid crystal phase transition. In the nematic phase ordering can be well described by the nematic director field $\vec{n}$ (SBF) and the uniaxial nematic orientational order parameter *S* (OPF). An example of a continuous translational symmetry breaking represents the nematic-smectic phase transition. In the smectic A phase the ordering is typically described by the phase factor *φ* (SBF) and the translational order parameter *η* (OPF).

For illustration purposes we consider orientational ordering in the nematic phase in the remaining part of this Section. We first show that a domain-type pattern in the SBF is inevitably formed at least temporally in the lower symmetry configuration after a quench from the higher symmetry phase even in absence of disorder. Then we give evidences that such domain pattern could be stabilized by impurities, which impose a quenched random field-type of disorder. In both cases the domain patterns are well described by a single characteristic length. We determine regimes where such patterns are expected.

#### Domain Coarsening Following a Fast Enough Phase Transition

We consider the onset of orientational ordering in the thermotropic nematic phase which is reached via the temperature quench from the isotropic phase in absence of static disorder. Immediately after the quench in causally disconnected parts of the system, a random value of SBF is chosen depending on a local preference mediated by a fluctuation [75, 76]. The resulting nematic structure is strongly orientationally frustrated and consequently spatially dependent variations in degree of ordering appear. The established configuration is extremely energetically costly and consequently it undergoes coarsening dynamics towards the uniform equilibrium nematic configuration. In this process the dominant role is played by *topological defects* [19]. These refer to regions in space where SBF is not uniquely defined. Defects can be treated as objects carrying a topological charge which is a conserved quantity [19, 22, 77]. In a bulk system one can get rid of defects only via mutual annihilation [78, 79] of their topological charges. The region, in which apparent deviations from the bulk ordering exist due to the presence of a defect is referred to as the *core* of defect [22, 74]. Its linear dimension is roughly given by the correlation length *ξ*. The core typically exhibits a different phase with higher energy density with respect to the surrounding phase.

The coarsening dynamics following the quench evolves via three qualitatively different stages. Immediately after the quench the *early stage* is entered in which the orientational order parameter *OPF ≡ S* exhibits an exponential growth [4, 76]. The *early stage* terminates roughly at the so called Zurek time [4], at which *S ∼ S_eq_*, where *S_eq_* determines the equilibrium value of *S*. At this stage topological defects are formed and consequently domains become visible [4]. Then the *domain regime* is entered [76]. In it the domain-type pattern in SBF is clearly pronounced. The domain pattern is well characterized by a single domain length *ξ_d_*(*t*). The subsequent domain growth gradually enters the dynamic scaling regime, where the power law *ξ_d_*(*t*) ∝ *t^γ^*is obeyed [75, 76]. The universal scaling coefficient *γ* strongly depends whether a conservation law for the order parameter exists or not [75]. The order parameter evolution across a domain wall obeys the *geodesic rule*, *i.e.,* it follows the shortest possible path in the order parameter space [4]. The domain growth is enabled by annihilation of topological defects. After some time the *late stage regime* [76] is entered in which the pattern appearance is dominated by individual topological defects. Finally, in bulk samples the defectless homogeneous structure is reached.

The above described mechanism of domain formation is universal and is referred to as the Kibble–Zurek mechanism [4, 80]. It was originally developed to explain coarsening dynamics in the early universe [80]. The only essential ingredients of this mechanism are (i) continuous symmetry breaking and (ii) causality. It has been shown that the size $\xi_{d}^{\left(0\right)}$ of *protodomains*, which are first formed at the Zurek time, depends on the quench rate. For example, for the temperature driven 2nd order phase transitions at *T* = *T_c_* the scaling law

(30) $$\xi_{d}∼\xi_{0}{\left(\frac{\tau_{Q}}{\tau_{0}}\right)}^{\upsilon /(1+\eta )}$$

is obeyed [4]. Here *τ_Q_* stands for the quench rate. The universal coefficients *υ* and *η* describe critical behavior of the relevant order parameter correlation length *ξ ∼ ξ*_0_ |*r*|*^−υ^* and the order parameter relaxation time *τ ∼ τ*_0_ *|r|^−η^* close to the transition as a function of the reduced temperature *r* = (*T − T_c_*)/*T_c_*. The quantities *ξ*_0_ and *τ*_0_ are bare relaxation length and relaxation time estimating size of *ξ* and *τ* at relatively low temperatures. The scaling law (30) is obeyed only for fast enough quenches. Theory [81] suggests that the condition

(31) $$\tau_{Q}/\tau_{0}|1-T_{G}/T_{c}|<\pi^{4}$$

should be obeyed, where *T_G_* stands for the Ginzburg temperature.

#### Imry-Ma Domain Pattern

Next, we estimate characteristic size *ξ_d_* of domains that are stabilized by random field type of disorder following original ideas of Imry and Ma [12]. The estimate is based on static ground, *i.e.,* it is not influenced by the history of a sample. For illustration purposes we consider the nematic LC phase perturbed by a random anisotropy-type of disorder. To estimate *ξ_d_* we balance the elastic free energy costs and free energy gains if the system obeys the imposed random field tendencies. Roughly speaking, if the domains are too small, the system possesses a large number of boundaries, whose energy is unfavorable. But if they are too large, they cannot order locally to take advantage of the local random fields. The compromise is a universal domain pattern, which is characterized by short range ordering.

We express the elastic free energy and the random anisotropy field density [17, 71] costs as $f_{e}∼\frac{K}{2}|\nabla \vec{n}{|}^{2}$ and $f_{RA}∼-\frac{w}{2}P_{2}(\vec{n}\cdot \vec{e})$, respectively. Here we assume that the unit vectors $\vec{e}(\vec{r})$ are spatially randomly orientationally distributed and *w* measures the random anisotropy field strength. We assume that the system breaks up into a domain pattern characterized by *ξ_d_*. Typical elastic distortion in an average domain is estimated by $〈f_{e}〉∼\frac{K}{2\xi_{d}^{2}}$, where < ... > denotes spatial average within a domain. In order to estimate 〈*f_RA_*〉 we set that inside the domain $\vec{e}(\vec{r})$ undergoes random spatial variations. We label an average distance, on which $\vec{e}$ significantly changes, by *ξ_r_* and estimate the averaging rate within this domain. On traversing a domain volume LC molecules experience [12, 64]

(32) $$N_{d}∼{\left(\frac{\xi_{d}}{\xi_{r}}\right)}^{d}$$

random alternations in $\vec{e}$ orientation. We refer to *N_d_* as the averaging number. For a large enough domain, where $\vec{n}$ is essentially homogeneously oriented along a symmetry breaking direction, the term $〈P_{2}(\vec{n}\;.\;\vec{e})〉$ averages to zero. According to the central limit theorem the averaging effectiveness is proportional to $1/\sqrt{N_{d}}$. Therefore one expects

(33) $$〈P_{2}(\vec{n}.\vec{e})〉∼\frac{1}{\sqrt{N_{d}}}∼{\left(\frac{\xi_{r}}{\xi_{d}}\right)}^{d/2}$$

Balancing free energy costs 〈*f_RA_*〉 and 〈*f_e_*〉 yields the Imry-Ma scaling law [12]

(34) $$\xi_{d}\propto \;w^{\frac{2}{d-4}}$$

#### Simulation Results

The Imry–Ma estimation of *ξ_d_* is based on statical grounds reflecting the balance between the elastic and random field free energy penalties. On the other hand the Kibble–Zurek mechanism suggest that the size of protodomains depends on the quench rate. It is known that impurities could pin [82] regions exhibiting relatively strong elastic distortions. Therefore, in a configuaration reached via a phase transition quench the (quasi) stable pattern migh depend on the relative size of protodomains and the average distance among neighboring impurities. The size of protodomains depends on the quench rate [4] and consequently the domain pattern could depend on history of samples. Note that there have been published contradicting results related to the fate of low symmetry phase in presence of disorder. Some authors predict presence of Imry–Ma clusters [12, 83], and others algebraic decay of correlations [2].

For this purpose [62, 84, 85], a systematic analysis have been recently carried out focusing on the impact of system history on randomly perturbed nematic phase. Simple minimal models in *d* = 2 and *d* = 3 were used in order to test universality of the Imry–Ma scaling prediction. Due to time consuming simulations and relatively wide phase space to be explored most simulations were realized at *T* = 0. Some predictions of these simulations were then tested at finite temperatures to confirm validity of obtained results for more realistic conditions. In the following we present the main results of these studies.

**The Imry–Ma scaling and memory effects at temperature zero :** The minimal model used is the following. Consider an ensemble of *N* cylindrically symmetric particles within a cubic lattice of unit lattice size *a*_0_. Local orientational ordering of a particle at the i-th site is given by an unit vector ${\vec{S}}_{i}$, to which we refer as a spin. The ± ${\vec{S}}_{i}$ orientations are equivalent in order to mimic mesoscopic symmetry of LC molecules. The spins experience a random anisotropy field enforced by impurities, the concentration of which is labeled by *p*. The impurities are randomly distributed within the lattice and locally force spins to orient along ${\vec{e}}_{i}$. The orientational distribution of ${\vec{e}}_{i}$ is isotropic.

The interaction energy *F* of the system is expressed as

(35) $$F=-\frac{1}{2}\sum J_{ij}{\left({\vec{S}}_{i}\cdot {\vec{S}}_{j}\right)}^{2}-\frac{1}{2}\sum w_{i}{\left({\vec{S}}_{i}\cdot {\vec{e}}_{i}\right)}^{2}-\frac{1}{2}\sum B^{2}{\left({\vec{S}}_{i}\cdot {\vec{e}}_{B}\right)}^{2}$$

The short range interaction *J_ij_* is equal to *J >* 0 for the first neighbors and is set to zero otherwise. It tends to orient spins parallel or antiparallel. At randomly chosen sites (their number is equal to *Np*) a finite anchoring strength is imposed *w_i_* = *w*, representing impurities. At the remaining sites it holds *w_i_* = 0. The last term takes into account presence of an ordering external electric or magnetic field $\vec{B}=B{\vec{e}}_{B}$. The spins are parametrized as ${\vec{S}}_{i}={\vec{e}}_{x}S_{i}^{\left(x\right)}+{\vec{e}}_{y}S_{i}^{\left(y\right)}+{\vec{e}}_{z}S_{i}^{\left(z\right)}$. Here ${\vec{e}}_{x}$, ${\vec{e}}_{y}$, and ${\vec{e}}_{z}$ represent unit vectors of the Cartesiam coordinate system {*x, y, z*} and the constraint $\left|{\vec{S}}_{i}\right|=1$ is imposed. Periodic boundary conditions are implemented.

In simulations one originates either from spatially randomly distributed orientations of spins, or from a homogeneously aligned sample along a single direction. We refer to these system histories as the *random* and *homogeneous initial condition*, respectively. The random initial configuration simulates sudden temperature quench from the isotropic phase. The homogeneous initial configuration can be realized by applying first a strong homogeneous external electric or magnetic field in the nematic phase. After a well enough alignment is achieved the field is switched off.

A system configuration is obtained by minimizing *F* with respect to spin components [84]. Therefore, the role of thermal fluctuations is neglected. Such an assumption is sensible deep in the nematic phase. In order to check validity of the Imry–Ma theorem *d* = 2 (2D) and *d* = 3 (3D) systems are considered. In 2D the simulations are constrained to the {*x, y*} plane and one sets $S_{i}^{\left(z\right)}=0$.

From obtained configurations the orientational correlation function *G*(*r*) is calculated. In 2D and 3D it is defined as

(36) $$G_{2D}(r)=〈2{\left({\vec{S}}_{i}\cdot {\vec{S}}_{j}\right)}^{2}-1〉$$

(37) $$G_{3D}(r)=\frac{1}{2}〈3{\left({\vec{S}}_{i}\cdot \vec{S_{j}}\right)}^{2}-1〉$$

respectively. The brackets denote the average over all lattice sites that are separated for a distance *r*. If the spins are completely correlated (*i.e.,* homogeneously aligned along a symmetry breaking direction) it follows *G*(*r*) = 1. On the other hand *G*(*r*) = 0 reflects completely uncorrelated spins.

In order to obtain structural details from calculated dependencies *G*(*r*) the ansatz

(38) $$G(r)=(1-s)e^{-{\left(r/\xi_{d}\right)}^{m}}+s$$

is used, where *ξ_d_*, *m*, and *s* are adjustable parameters. The quantity *ξ_d_* estimates the average domain length (the coherence length) of the system. Over this length the spins are relatively well correlated. The distribution width of *ξ_d_* values is measured by *m*. Dominance of a single coherence length in the system is signaled by *m* = 1. A magnitude and system size dependence of *s* reveals the degree of ordering within the system. The case *s* = 0 indicates the short range order (SRO). A finite value of *s* reveals either the long range order (LRO) or quasi long range order (QLRO). To distinguish between these two orderings a finite size analysis should be carried out. If *s*(*N*) saturates at a finite value the system exhibits LRO. If *s*(*N*) exhibits algebraic dependence on *N* the system possesses QLRO.

**Figure 5.**
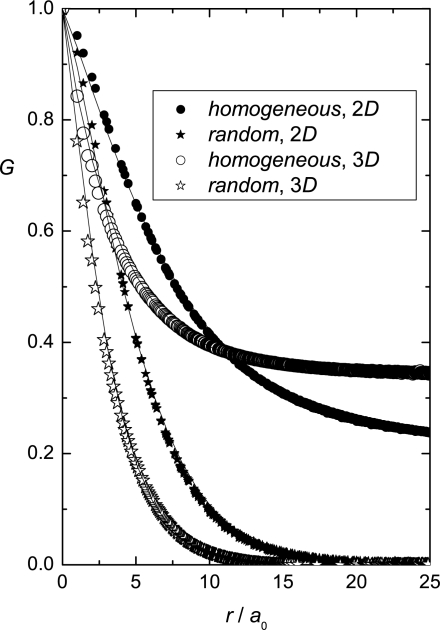
*G*(*r*) for homogeneous and random initial configurations in 2D and 3D systems, *p* = 0.3, *w* = 2. Note the difference between saturated *G*(*r*) dependence for random initial conditions. In the random case the correlations vanish (*G*(*r → ∞*) = 0) and for homogeneous initial conditions *G*(*r* → ∞) reaches a finite plato.

Representative *G*(*r*) dependencies for the random and homogeneous initial conditions in 2D and 3D for *B* = 0 are shown Figure 5. For the random condition in all studied cases [85] SRO was obtained, *i.e., s* = 0. On the contrary configurations obtained from the homogeneous initial condition yield *s >* 0 at a fixed value of *N* for weak anchoring strengths *w* and low enough concentrations *p*. This suggests either LRO or QLRO.

To check the validity of the Imry–Ma scaling the obtained *ξ_d_* values were fitted with the ansatz

(39) $$\xi_{d}=\xi_{0}w^{-\gamma }+\xi_{\infty }$$

**Figure 6.**
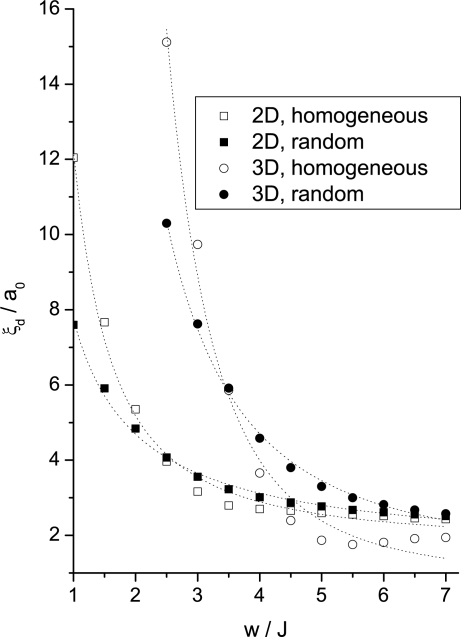
*ξ_d_*(*w*) variations for different initial configurations for 2D and 3D, *p* = 0.3. Only for the random initial configuration the Imry-Ma theorem is obeyed.

Results show that in the strong anchoring limit (*w >* 10) *ξ_d_* does not depend on the history of the system. In the weak anchoring regime the Imry–Ma scaling was perfectly obeyed only for random initial conditions. In 2D and 3D the simulations yield *ξ_d_* ∝ *w*^−1±0.15^ and *ξ_d_* ∝ *w*^−2±0.3^, respectively. On the contrary, for the homogeneous initial configurations significant departures from the Imry–Ma behavior are observed. In this case SRO is not obtained for weak enough anchoring conditions, although systems still reveal a characteristic length as already suggested by Giamarchi and Doussal [15]. The simulations in this case suggest [85] *ξ_d_* ∝ *w*^−1.6±0.1^ for 2D and *ξ_d_* ∝ *w*^−3.2±0.25^ for 3*D*, see Figure 6.

**Figure 7.**
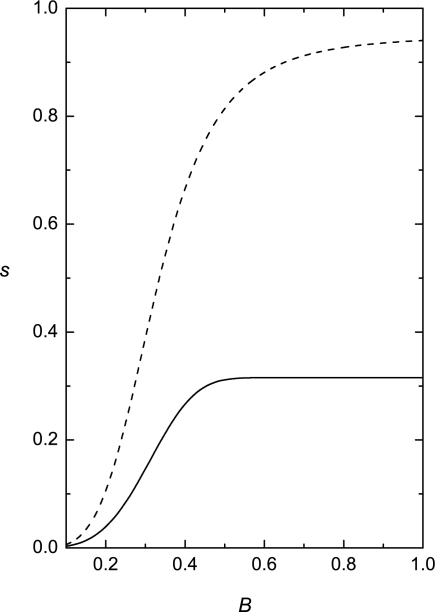
Value of *s* = *G*(*r* → ∞) as a function of system history for 2D (the dashed curve, *N* = 120 *×* 120) and 3D (the solid curve, *N* = 60 *×* 60 *×* 60), *p* = 0.25. The initial configurations were calculated in *B* = 0 using random initial configurations, yielding *s* = 0. Then the field *B* was switched on and the resulting (quasi) equilibrium configurations were calculated. Afterwards the field was switched off and again (quasi) equilibrium configurations were calculated, characterized by *s >* 0. Values of *s* reflect the magnetic histories of the samples.

It was further analyzed [84] behavior of systems using standard *zero field cooled* and *field cooled* cycles which are used in order to probe glassy features of systems. For this purpose simulations were started from random initial conditions. The zero field cooled configurations were calculated for *B* = 0. To obtain the field cooled configuration one first calculates metastable state in the presence of *B*. Afterwards *B* is switched off and again the quasiequlibrium state is searched for. The corresponding values of *s*, reflecting range of ordering in these cycles, are presented in Figure 7 in 2D and 3D. In both cases qualitatively similar behavior is obtained. Note that the zero field cooled configurations yield *s* = 0. One sees that the field cooled configuration possess a finite value of *s*. With increasing value of the external field, *s* in the first stage gradually increases and then saturates at a fixed value. These simulations signal pronounced memory effects in systems of interest.

**Finite temperatures :** In order to verify memory dependent size of domains at finite temperatures a Brownian molecular dynamic study [76, 86] was performed [62]. The domain patterns formed by rodlike particles, representing a LC molecule, was studied as a function of the concentration *p* of impurities imposing a random anisotropy field-type disorder to the particles and the history of the samples. The interaction between the neighboring spins ${\vec{S}}_{i}$ is given by the modified Lebwohl–Lasher potential [87]

(40) $$f_{ij}=\frac{J}{r^{6}}{\left({\vec{S}}_{i}.\;{\vec{S}}_{j}-\frac{3\eta }{r^{2}}({\vec{r}}_{ij}.\;{\vec{S}}_{i})({\vec{r}}_{ij}.\vec{\;S_{j}})\right)}^{2}.$$

Here $r=\left|{\vec{r}}_{ij}\right|=\left|{\vec{r}}_{i}-{\vec{r}}_{j}\right|$ denotes the separation between the *i*-th and *j*-th spin in the 3D cubic lattice, *J* is a positive interaction constant and the parameter *η* describes the degree of orientational anisotropy. The case *η* = 0 is equivalent to the Lebwohl–Lasher [88], also referred to as the Maier–Saupe lattice model, which corresponds in the continuum limit to the approximation of equal Frank nematic elastic constants [42]. For *η* = 1 one gets the induced-dipole–induced-dipole type potential. However, the interaction *f_ij_* yields the nematic-like properties only for *η <* 0.3 [87]. Despite its simplicity, the Lebwohl–Lasher model well mimics main static and dynamic properties of a typical isotropic-nematic (I-N) phase transition. By studying cases *η >* 0 one probes the impact of elastic anisotropies on the system properties.

The system is enclosed within a cube of volume $Na_{0}^{3}$, where *N* is the number of molecules in the system and *a*_0_ is the characteristic size of the unit cubic cell of the lattice. At the systems’ boundary the periodic boundary conditions are imposed. Impurities of concentration *p* are assigned to randomly chosen lattice sites ${\vec{r}}_{i}^{\left(0\right)}$ of the system, where the unit cell is the simple cubic one with the lattice constant *a*_0_. It is assumed, that the *i*-th impurity enforces the orientation ${\vec{e}}_{i}$ to the neighboring *j*-th spin via the short range potential

(41) $$f_{ij}=-\frac{w}{r^{6}}{\left({\vec{e}}_{i}.\;{\vec{S}}_{j}-\frac{3\eta }{r^{2}}({\vec{r}}_{ij}.\;{\vec{S}}_{i})({\vec{r}}_{ij}.\vec{\;S_{j}})\right)}^{2}$$

The orientations of unit vectors ${\vec{e}}_{i}$ are randomly distributed in 3*D*, and *w* stands for the orientational anchoring constant. The interaction energy *W_int_* of the whole sample is given as a sum over all pair interactions. In calculations one limits to the interactions with neighbors within a sphere of radius 2*a*_0_,

The positions ${\vec{r}}_{i}$ of molecules are allowed to fluctuate about the lattice points ${\vec{r}}_{i}^{\left(o\right)}$ of the three dimensional lattice. In such a way one gets rid of the lattice induced ordering anisotropy which is known to appear by choosing the unite cubic cell. The random departures $\Delta r=|\Delta {\vec{r}}_{i}(t)|=\left|{\vec{r}}_{i}-{\vec{r}}_{i}^{\left(o\right)}\right|$ obey the Gaussian statistics centered at Δ*r* = 0, the width of which depends on the temperature *T*.

The orientation of the *i*-th spin is parameterized in the laboratory frame as

(42) $${\vec{S}}_{i}=(\text{sin}\;\vartheta_{i}\text{cos}\;\phi_{i},\text{sin}\vartheta_{i}\text{sin}\phi_{i},\text{cos}\vartheta_{i})$$

where $\vartheta_{i}=\vartheta ({\vec{r}}_{i},t)$ and $\phi_{i}=\phi ({\vec{r}}_{i},t)$ represent dynamic variables of the model. The rotational dynamics of the system is driven by the Brownian molecular dynamics [76, 86]. At each time interval Δ*t* (one sweep) the molecular orientation at the *i*-th site is updated in the local frame ${\vec{r}}^{\prime }=(x^{\prime },\;y^{\prime },\;z^{\prime })$ obeying the equations [86]

(43) $$\vartheta_{i}^{\left(x^{\prime }\right)}=-\frac{D\Delta t}{k_{B}T}\sum_{j\neq i}\frac{\partial f_{ij}}{\partial \vartheta_{j}^{\left(x^{\prime }\right)}}+\vartheta_{r,i}^{\left(x^{\prime }\right)}$$

(44) $$\vartheta_{i}^{\left(y^{\prime }\right)}=-\frac{D\Delta t}{k_{B}T}\sum_{j\neq i}\frac{\partial f_{ij}}{\partial \vartheta_{j}^{\left(y^{\prime }\right)}}+\vartheta_{r,i}^{\left(y^{\prime }\right)}$$

in which the orientational diffusion tensor is diagonal. Its eigenvalues are assumed to be degenerate, equal to *D*, and *k_B_* is the Boltzmann constant. The *z′*-axis of the local frame is oriented along the long axis of the LC molecule. The angles $\vartheta_{i}^{\left(x^{\prime }\right)}$ and $\vartheta_{i}^{\left(y^{\prime }\right)}$ correspond to small rotations of the *i*-th molecule about the *x′* and *y′* axis, respectively. The gradient of the potential for these two rotations is calculated numerically. The quantities $\vartheta_{r,i}^{\left(x^{\prime }\right)}$ and $\vartheta_{r,i}^{\left(y^{\prime }\right)}$ are stochastic variables obeying the Gaussian probability distribution centered at $\vartheta_{r,i}^{\left(x^{\prime }\right)}=\vartheta_{r,i}^{\left(y^{\prime }\right)}=0$, where the distribution widths $\Delta \vartheta_{r}^{\left(x^{\prime }\right)}=\Delta \vartheta_{r}^{\left(y^{\prime }\right)}$ are proportional to $\sqrt{T}$ [86]. The corresponding multiplicative constant is chosen such to yield a correct equilibrium value of the nematic uniaxial order parameter *S* in the continuum picture. The shortest time interval Δ*t* of the model in the simulation is set by Δ*tD ∼* 0.01. For a typical nematic LC [42] this ranges within the interval Δ*t ∼* 0.001 *μs* to Δ*t ∼* 0.1 *μs* depending on the size of a molecule (*i.e.,* in our model a molecule in fact corresponds to a cluster of *real* molecules).

In the simulations the average domain size was monitored which is estimated from a pattern as follows. One calculates an average volume *V_d_* in which relatively small changes occur in the orientational ordering. As the criterion of being in a domain the condition $\left|{\vec{m}}_{i}\;\cdot \;{\vec{m}}_{j}\right|>1-\Delta$ is imposed, where the *i*-th and *j*-th spin are adjacent. A value Δ *∼* 0.2 is chosen corresponding to the amplitude of thermal fluctuations at approximately double width of the Gaussian distribution. The domain size is then estimated via $\xi_{d}∼{\left(\frac{3V_{d}}{4\pi }\right)}^{1/3}$.

**Figure 8.**
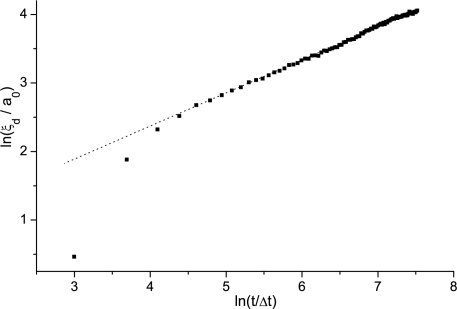
The time evolution of the characteristic domain length *ξ_d_* in a bulk sample following the sudden quench from the isotropic phase. Soon after the quench the scaling law *ξ_d_* *∝ t*^0.49^ is obeyed, which is plotted with the dotted line. *N* = 70^3^, *η* = 0.

**Figure 9.**
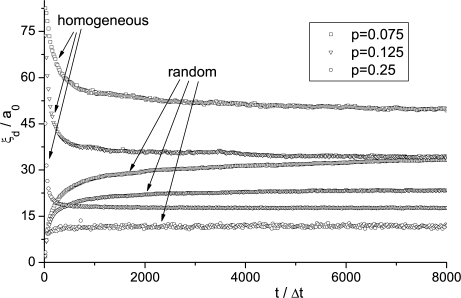
The domain growth for different concentrations of impurities after sudden quench from isotropic phases or homogeneously aligned structures. *N* = 70^3^, *η* = 0.

**Figure 10.**
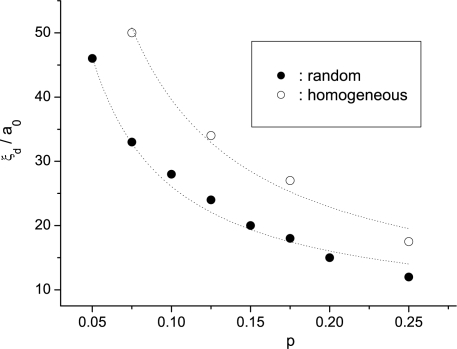
Saturated average domain length values as a function of *p* and history of the samples. *N* = 70^3^, *η* = 0.

In Figure 8 the time evolution of the domain coarsening in the pure bulk sample (*i.e., p* = 0) is shown following a sudden isotropic-nematic phase transition. Soon after the quench the scaling regime is entered, where *ξ_d_*(*t*) ∝ *t*^0.49^. Simulations show negligible influence on *η*. The domain length time evolution *η_d_*(*t*) for different concentrations *p* for different histories of systems is shown in Figure 9. The system was either quenched (i) from the isotropic phase, and (ii) from perfectly homogeneously aligned sample. The latter case corresponds to a sudden switch-off of a strong external magnetic or electric ordering field. For these histories the *ξ_d_*(*t*) values monotonously (i) increase and (ii) decrease with time, respectively. The saturated values *ξ_d_* of patterns are shown in Figure 10. They exceed the average distance between impurities and show significant memory dependence in line with predictions obtained using much simpler approach described in the previous subsection. The patterns originated from isotropic configurations have shorter values of *ξ_d_* in comparison to the structures reached from homogeneously aligned samples. In all cases the scaling *ξ_d_* ∝1/*p* is well obeyed. This observation in line with experimental results obtained in mixtures of nematic LC molecules and aerosil particles [83], for which the model described is appropriate.

### 4.2. Phase Behavior

In the following we assume that the random anisotropy field gives rise to a domain type pattern. We analyze phase expected behavior for perturbed nematic LC and nematic-non nematogen mixture.

#### Phase behavior of RAN

The random anisotropy nematic type disordering field enters the expression for the free energy *F* (18) via the surface contribution *f_s_* (26). In the case of a liquid crystal confined in a porous medium or in a mixture composed of liquid crystal and a second component (impurities, or colloids, or carbon nanotubes), due to essentially randomly spatially varying liquid crystal-substrate interface orientation, the easy axis ${\vec{e}}_{s}$ exhibits random spatial variations. If a relatively weak disorder is enforced to a liquid crystal phase, then according to the Imry-Ma prediction the competition between the ordering and random field disordering tendencies can result in a domain-like pattern [12]. Accepting this prediction, according with the central limit theorem, the surface contribution to the free energy becomes:

(45) $$f_{s}=-\frac{W}{2}S〈{\left(\vec{n}\cdot {\vec{e}}_{s}\right)}^{2}-\frac{1}{3}〉∼-\frac{W}{2}S\frac{1}{\sqrt{N}}∼-\frac{W}{2}S{\left(\frac{\xi_{r}}{\xi_{d}}\right)}^{3/2}$$

where *ξ_d_* is the average linear length of the domain, *ξ_r_* is a typical scale on which the easy axis undergoes random spatial variations, and *N ∼* (*ξ_d_*/ξ*_r_*)^3^. Note that *ξ_d_* is also the length on which a typical director field distortions are expected to evolve if other external fields are absent. On the contrary locally induced perturbations in *S* are expected to take place at the liquid crystal-perturber interface on the nematic correlation length scale *ξ_n_*(*T*), which is a microscopic quantity (typically in the range of nm). Therefore in the mesoscopic limit we can consider only the spatial variations of the director $\vec{n}$, and for a distorted nematic one typically expects that the elastic free energy density is given by

(46) $$f_{e}=\frac{1}{2}\frac{LS^{2}}{\xi_{d}^{2}}$$

For numerical purposes we introduce instead the length $\xi =\sqrt{\xi_{d}^{2}-\xi_{r}^{2}}$. Therefore the case *ξ* = 0 corresponds to *ξ_d_* = *ξ_r_*.

Taking into account these previous considerations, the free energy density can now be written as

(47) $$f=\frac{3}{2}a(T-T*)S^{2}-\frac{3}{4}BS^{3}+\frac{9}{4}CS^{4}+\frac{L}{2\xi_{r}^{2}(1+\xi^{2}/\xi_{r}^{2})}S^{2}-\frac{W}{2\xi_{d}{\left(1+\xi^{2}/\xi_{r}^{2}\right)}^{3/4}}S$$

For convenience we introduce nondimensional quantities. The temperature is replaced by the reduced temperature *τ* = (T − T*)/(*T_NI_* − T*) where *T_NI_* = T* + *B*^2^/24*aC*. In this new temperature scale *τ_NI_* = 1, τ* = 0, and *τ*^+^ = 9/8. The nematic order parameter is normalized with respect to its value at the nematic-isotropic phase transition of the homogeneous system *S̅* = 6*CS/B* and the nondimensional free energy density is defined as *f̄* = 24^2^*C*^3^*f/B*^4^. The nondimensional length, elastic constant and surface anchoring are given by *ξ̄* = *ξ/ξ_r_* and $\bar{L}=8CL/B^{2}\xi_{r}^{2}$ and Λ = 48*C*^2^*W/ξ_d_B*^3^.

Omitting the bar notation, the dimensionless free energy density is written in the following form:

(48) $$f=\tau S^{2}-2S^{3}+S^{4}+\frac{L}{1+\xi^{2}}S^{2}-\frac{\Lambda }{{\left(1+\xi^{2}\right)}^{3/4}}S$$

Taking the limit *ξ → ∞* in Equation (48) the undistorted bulk free energy density of the nematic phase is reproduced. The finite value of *ξ* reflects the competition between the *elastic term* $\frac{L}{1+\xi^{2}}S^{2}$ (favoring *ξ → ∞*) and the *surface field term* $-\frac{\Lambda }{{\left(1+\xi^{2}\right)}^{3/4}}S$ (favoring *ξ →* 0).

Minimizing Equation (48) with respect to *ξ* leads to

(49) $$\xi =\{\begin{array}{ll} 0 & \text{when}\;S<\;S_{c}\\ {\left({\left(\frac{S}{S_{c}}\right)}^{4}-1\right)}^{\frac{1}{2}} & \text{when}\;S>\;S_{c} \end{array}$$

where *S_c_* = 3Λ/4*L*. The corresponding free energy density is given by

(50) $$f=\{\begin{array}{ll} f_{P}=\tau S^{2}-2S^{3}+S^{4}-\Lambda S+LS^{2} & \text{when}\;S<S_{c}\\ f_{N}=\tau S^{2}-2S^{3}+S^{4}-\frac{27}{256}\Lambda^{4}L^{-3}S^{-2} & \text{when}\;S>S_{c} \end{array}$$

The subscripts *P* and *N* stand for the *paranematic* and *speronematic* ordering, respectively. The paranematic phase closely resembles the isotropic phase but exhibits a finite degree of nematic ordering. The speronematic phase represents a distorted nematic phase, *i.e.,* the phase with a finite value of *ξ*.

**Figure 11.**
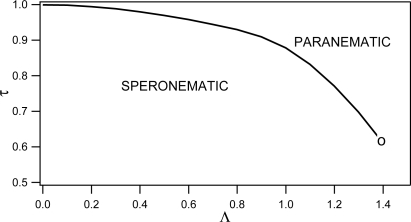
The phase diagram (Λ*, τ*) calculated for *L* = 1. The discontinuous paranematic-speronematic phase transition is marked with the full line and the circle at (*τ_c_* = 0.62*,* Λ*_c_* = 1.39) marks the tricritical point.

The (Λ*, τ*) phase diagram for *L* = 1 is shown in Figure 11.

For a weak enough disorder (Λ *<* Λ*_c_* = 1.39) there is a first order phase transition from a paranematic phase (minimum of *f_P_*) to a speronematic phase (minimum of *f_N_*) at the critical temperature *τ* = *τ_c_* = 0.62. A continuation of this line to higher values of Λ *>* Λ*_c_* (not shown in Figure 11) marks a continuous transition characterized by a discontinuity in the slop *dS/dτ*. Note that at this line the switching between the two solutions for *ξ* (49) takes place. In the literature we have not found any experimental evidence resembling the observed slope discontinuity. Therefore, we believe that this anomaly is probably an artifact of our method. In reality a gradual evolution of the nematic ordering is expected.

**Figure 12.**
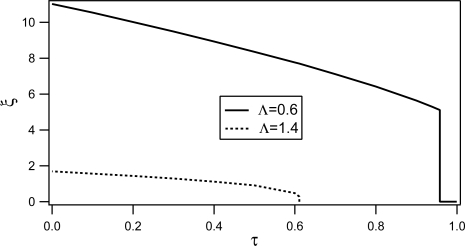
*ξ*(*t*) dependence for *L* = 1.

**Figure 13.**
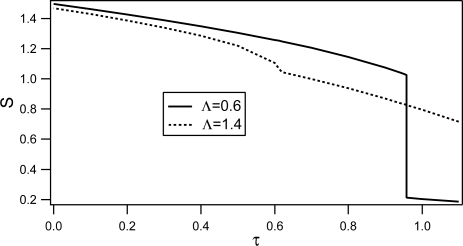
*S*(*τ*) dependence for *L* = 1.

The behavior of the correlation length *ξ* and of the order parameter *S* as functions of reduced temperature *τ* are shown in Figures 12 and 13 respectively.

For low values of the order parameter *S* (*i.e., S < S_c_*), the length *ξ* is zero and the director orientations are correlated only on *ξ_r_*. At larger values of *S* the cost of changing the orientation from point to point is increased. The system responds by increasing the length scale *ξ* over which the director orientations are correlated. In the low disorder limit Λ *→* 0, the true nematic phase with infinite *ξ* is recovered. We mention that *ξ* is a quantity of key importance for understanding the phase transition in the presence of disorder. It is in fact a direct measure of *resistance* or the *stability* of the nematic phase in the presence of quenched disorder. In principle, *ξ* could be larger than the typical length over which the disordered structure is correlated. As expected, *ξ* increases as *τ* decreases, and indeed as *S* increases.

The profiles of the order parameter for two values of Λ shown in Figure 13 indicate that the bulk first order nematic-isotropic phase transition is weakened and eventually suppressed by increasing randomness.

#### Nematic-non-Nematic Mixture

In this section we present some results concerning the influence of a random anisotropy type disorder on the phase separation of the nematogen–non-nematogen mixture. We assume that the impurities (*i.e.,* the non-nematic component) via the impurity–liquid crystal interface orientational anchoring interaction enforce to the liquid crystal phase a kind of random anisotropy field [72]. A combination of the random anisotropy nematic model and Flory–Huggins [89] model is used for the theoretical study of the phase stability of the mixture as a function of the absolute temperature *T*, impurity volume fraction Φ, and random anisotropy field strength Λ. We show that the random anisotropy field qualitatively changes the topology of the (Φ, *T*) phase diagram.

The mixture is characterized by the volume fractions of the two components:

(51) $$\Phi_{i}=\frac{N_{i}\upsilon_{i}}{\sum_{i=1}^{2}N_{i}\upsilon_{i}}\;\text{with}\;\sum_{i=1}^{2}\Phi_{i}=1$$

where *N_i_* is the number of molecules of component *i* (*i* = 1 defines the liquid crystal and *i* = 2 the non-nematogenic fluid) and *v_i_* is the volume of a particle of component *i*. In what follows, we consider that the volumes of a nematic and impurity molecule are equal (*υ*_1_ = *υ*_2_ = *υ*) and Φ_2_ = Φ (due to the conservation of the number of particles Φ_1_ = 1 *−* Φ). The orientational order of the mixture is characterized by the nematic order parameter *Q_αβ_* which, in the case of uniaxial nematic state, is given by Equation (21).

The free energy *F* of the system is expressed as

(52) $$F=\int \left[f_{m}(\Phi )+(1-\Phi )(f_{h}(\Phi ,Q_{\alpha \beta })+f_{e}(Q_{\alpha \beta ,\gamma })\right]\;dV+(1-\Phi )\int f_{s}dA$$

where *V* is the volume of the system and *A* is the surface area of the liquid crystal–impurity interface.

The first term is the free energy density of the isotropic mixing for the two components [89]

(53) $$f_{m}(\Phi )=\frac{Nk_{B}T}{V}[(1-\Phi )\text{ln}\;(1-\Phi )+\Phi \text{ln}\Phi +\chi \Phi (1-\Phi )]$$

where *k_B_* is the Boltzmann constant and *χ* = (*U*_0_/*k_B_T*) is the Flory–Huggins interaction parameter related to isotropic interaction between unlike molecular species.

The second term in Equation (52) is the Landau–de Gennes free energy density of the isotropic-nematic phase transition generalized to include the interaction between nematic and non-mesogenic molecules:

(54) $$f_{h}(\Phi ,Q_{\alpha \beta })=a[T-(1-\lambda \Phi )T*]Q_{\alpha \beta }Q_{\beta \alpha }-BQ_{\alpha \beta }Q_{\beta \gamma }Q_{\gamma \alpha }+C{\left(Q_{\alpha \beta }Q_{\beta \alpha }\right)}^{2}$$

The coupling between Φ and *Q_αβ_* in Equation (54) results from microscopic considerations [62, 90]).

The elastic free energy density *f_e_* and the anchoring term *f_s_* have been discussed in sections (3.2.) and (4.2.) and are given by Equations (23) and (24) respectively.

Using the non-dimensional variables defined in section (4.2.) the dimensionless free energy density is given by

(55) $$\begin{array}{l} f=\Gamma [(1-\Phi )\text{ln}\;(1-\Phi )+\Phi \text{ln}\Phi +\chi \Phi (1-\Phi )]+(1-\Phi )[(\tau +\lambda \Phi )S^{2}-2S^{3}+S^{4}]\\ \;\;\;\;\;\;\;\;+(1-\Phi )\;\left[\frac{L}{1+\xi^{2}}S^{2}-\frac{\Lambda \Phi }{{\left(1+\xi^{2}\right)}^{3/4}}S\right] \end{array}$$

where Γ = 24^2^*Nk_B_TC*^3^/*V B*^4^.

Note that the local ordering tendency of impurities induces a finite value of *S* even in the isotropic phase. One commonly refers to such the case as the *paranematic* ordering. The equilibrium condition at the nematic-paranematic phase transition is given by the following set of Equations [91]:

(56) $$\Delta g(\Phi ,S)=0;\frac{\partial \Delta g}{\partial \Phi }(\Phi ,S)=0;\frac{\partial \Delta g}{\partial S}(\Phi ,S)=0$$

where

(57) $$\Delta g(\Phi ,S)=f(\Phi ,S)-f(\Phi_{p},S_{p})-\mu (\Phi -\Phi_{p})$$

is the difference in grand potential density between the two phases and

(58) $$\mu =\frac{\partial f}{\partial \Phi }(\Phi_{p},S_{p})$$

is the chemical potential. The ordering in the nematic and paranematic phase is determined by pairs (Φ*, S*) and (Φ*_p_, S_p_*), respectively.

**Phase behavior in the absence of randomness :** We first consider the phase behavior of the system in the absence of the random filed. In this case the elastic and surface terms are absent (*i.e., f_s_* = *f_e_* = 0 and Equation (55) becomes:

(59) $$f=\Gamma [(1-\Phi )\text{ln}\;(1-\Phi )+\Phi \text{ln}\;\Phi +\chi \Phi (1-\Phi )]+(1-\Phi )[(\tau +\lambda \Phi )S^{2}-2S^{3}+S^{4}]$$

**Figure 14.**
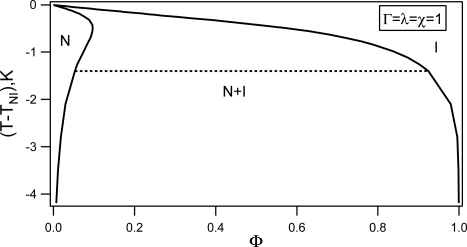
Phase diagram for nematic – non-nematic binary mixture in the absence of disorder for Γ = *λ* = *χ* = 1.

In this case the paranematic phase is replaced by the isotropic one and *S_p_* = *S_i_* ≡ 0, where *S_i_* stands for the degree of ordering in the isotropic phase. Taking into account Equations (56) we calculate a representative phase diagram of the nematic - impurity (*i.e.,* nematic– non-nematic) mixture, that is plotted in Figure 14 for Γ = *λ* = *χ* = 1. The solid curve refers to the binodal, that constitutes the actual phase boundary. For temperatures below *T_NI_* = 317.5K (value corresponding to pure 5CB [45]), there exists a two-phase coexistence region (N+I) between the isotropic (I) and nematic phase (N). On decreasing the temperature the biphasic region broadens.

To show how the onset of nematic ordering triggers phase separation in conventional liquid crystal, we rewrite *f* Equation (59)) in the following form

(60) $$f=\Gamma [(1-\Phi )\text{ln}\;(1-\Phi )+\Phi \text{ln}\;\Phi +\chi_{e\;f\;f}\Phi (1-\Phi )]+(1-\Phi )\;(\tau_{e\;f\;f}S^{2}-2S^{3}+S^{4})$$

where *τ_eff_* = *τ* and *χ_eff_* = *χ* + *λS*^2^/Γ stands for the effective Flory–Huggins interaction parameter. We see that the nematic ordering effectively increases the Flory–Huggins interaction parameter, that can potentially lead to order-induced phase separation.

**Phase behavior in the presence of random field:** In this section some theoretical results concerning the influence of disorder Λ *>* 0 on phase behavior of thermotropic nematic–non-nematic mixture are presented. The phase separation in the presence of random field takes place between the paranematic and speronematic phases. The paranematic phase closely resembles the isotropic phase but exhibits a finite degree of nematic ordering and *ξ* = 0. The speronematic phase represents distorted nematic phase that is characterized by a finite value of *ξ* (for an ordinary nematic phase *ξ → ∞*).

Minimizing Equation (55) with respect to *ξ* leads to

(61) $$\xi =\{\begin{array}{ll} 0 & \text{when}\;S<\;S_{c}\\ {\left({\left(\frac{S}{S_{c}}\right)}^{4}-1\right)}^{\frac{1}{2}} & \text{when}\;S>\;S_{c} \end{array}$$

where *S_c_* = 3ΛΦ/4*L*. The corresponding free energy density is given by,

(62) $$f=\{\begin{array}{ll} f_{P}=f_{m}(\Phi )+(1-\Phi )[(\tau +\lambda \Phi )S^{2}-2S^{3}+S^{4}-\Lambda \Phi S+LS^{2}] & \text{when}\;S<S_{c}\\ f_{N}=f_{m}(\Phi )+(1-\Phi )[(\tau +\lambda \Phi )S^{2}-2S^{3}+S^{4}-\frac{27}{256}\Lambda^{4}\Phi^{4}L^{-3}S^{-2}] & \text{when}\;S>S_{c} \end{array}$$

The subscripts *P* and *N* stand for the *paranematic* and *speronematic* ordering, respectively.

To calculate the phase diagram we use the equilibrium conditions given by the Equations (56). The solution of these equilibrium conditions is determined by four quantities: (i) the paranematic phase is determined by Φ*_p_* and *S_p_* and (ii) the speronematic phase by Φ*_s_* and *S_s_*.

**Figure 15.**
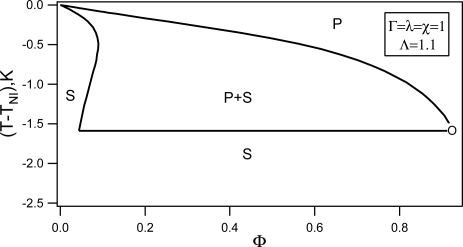
The influence of disorder on the phase separation for nematogen – non-nematogen binary mixture (see the main text).

The phase diagram (*T − T_NI_*, Φ) for Γ = *λ* = *χ* = 1 and Λ = 1.1 is shown in Figure 15.

In the vicinity of *T_NI_* the phase diagram is very similar to that in the absence of random field. But at some value of temperature *T − T_NI_* = *−*1.6K (which depends on the value of Λ), the paranematic order parameter does not fulfill the condition *S_p_ < S_c_* = 3ΛΦ/4*L*. As a consequence, the system is in only one state, the speronematic phase and correspondingly the phase separation cancels. This fact can have important consequences in the thermodynamic properties of the mixture, for example can explain the double pick in the specific heat experimentally observed in similar systems. This work is now in progress.

### 4.3. Experimental Observations

The described phenomenological RAN model roughly describes behavior of nematic LC experiencing a quenched random anisotropy field disorder. In the following we show some experimental evidences supporting this approach and discuss in which studied physical properties the influence of disorder might be significant for a temperature interval covering isotropic and nematic ordering in bulk LC samples.

In experimental studies the disorder is typically introduced geometrically by different perturbers via a varying LC–perturber interface [16]. For perturbers one commonly chooses inert porous matrices hosting LC phases or networks formed by aerosil [24, 25] particles in the aerosil–LC mixtures. As matrices aerogels and Controlled-Pore Glasses (CPG) [31] are conventionally used. Aerosil mixtures [26] are particularly useful because one can obtain qualitatively different random field-type regimes by changing the concentration of aerosil particles.

All these perturbers introduce into systems a new characteristic length scale *R*. In the case of aerosil nanoparticles this is the mean aerosil void [24] size *R* ∼ 2/(*aρ_s_*), where *ρ_s_* stands for the mass density of the aerosil particles and *a* is their active surface. In case of porous materials *R* represents a characteristic void radius. On commonly assumes that with decreasing value of *R* the disorder strength monotonously increases. However, a more detail analysis suggests that the dependence between *R* and the disorder strength is more complicated, at least in LC–aerosil mixtures [92]. Note that values of *R* are in most samples well below 1*μm*. In such systems, confinement, surface wetting and finite size effects could also play a significant role.

Most studies have been performed in mixtures of LC phases and aerosil nanoparticles. The spherular aerosil particles have diameter 2*R_s_* ∼ 7 *nm*. The specific surface area of aerosils [24, 26] is *a ∼* 300 *m*^2^/*g* for hydrophilic (type 300), and *a ∼* 280 *m*^2^/*g* for hydrophobic (type R812) aerosils. The neighboring aerosils could be linked via covalent bonds forming networks exhibiting moderate branching. By varying the concentration of aerosils *ρ_s_* three qualitatively different networks [24, 26] can be obtained. For *ρ_s_*< 0.01 *g/cm*^3^ the aerosil particles are more or less randomly distributed in the system. For *ρ_s_* > 0.01 *g/cm*^3^ the aerosils form a gel-like thixotropic network structure. In the so called *annealed regime*, ranging roughly between *ρ_s_* ∼ 0.01 *g/cm*^3^ and *ρ_s_* ∼ 0.1 *g/cm*^3^, the aerosil network is relatively responsive. It can rearrange in order to partially anneal the elastic stress imposed by the surrounding LC phase. In the *stiff regime*, corresponding to *ρ_s_* > 0.1 *g/cm*^3^, the aerosil network becomes a rigid-like, imposing a quenched type of disorder to surrounding LC molecules.

Several investigations have been also carried out in LC confined to aerogel matrices. Aerogels [27] form random networks of silica backbones in an open connected void space. The void pore size distribution is rather broad and the average characteristic pore size *R* can be far below 1 *μm*, depending on the density *ρ_a_* of the gel. The porosity of aerogels can be extremely high therefore providing host lattices of relatively high surface to volume ratio. The porosity can be varied between 0% and 98% by partial desification [93].

X-rays studies [24] reveal that the aerogel matrices and aerosil networks exhibit similar structures for high enough densities. Roughly equivalent structures obey equality [94] *ρ_s_ ∼ ρ _a_φ_p_* where *φ_p_* stands for the aerogel pore volume fraction. Furthermore, aerogels–LC and aerosil–LC mixtures are transparent and can be therefore studied also by means of optical spectroscopy.

Much work focusing on the influence of disorder on LC behavior has also been carried out in Controlled-pore glasses (CPG) [32, 95, 96]. A CPG matrix [32] consists of strongly curved and interconnected voids, that introduce a certain degree of randomness into the systems. Each void resembles a curved cylinder of radius *R* with a rather narrow distribution of *R*-values. Samples with radia from several *nm* up to *μm* can be obtained.

In all these samples the orientational anchoring condition at the perturber–LC interface depends on interface treatment and also on the type of LC used. However as a rule, LC–perturber interfaces tend to increase degree of LC ordering locally [96]. Consequently, *e.g.,* above the bulk I-N phase transition temperature *T_IN_* the isotropic phase is replaced by the paranematic phase, exhibiting a finite degree of nematic orientational ordering. On the other hand disorder gives rise to frustration in orientational ordering and therefore suppresses the degree of ordering. We will henceforth refer to the randomly frustrated nematic phase as the *speronematic* phase.

Regarding the range of ordering of the disordered phase, most studies confirm the prediction that the broken phase exhibits a domain-type short range order (SRO) in LC ordering. SRO was reported in samples using aerogels [3], CPGs [95] and aerosil particles [24] as LC perturbers. Most elaborate experimental investigations confirming the Imry–Ma hypothesis was performed by Bellini *et al.* [83] in LC–aerosil mixtures. Note that several analytic studies predict that quasi long range ordering is established instead or even LRO for weak enough degree of disorder [2]. However, these predictions have a relatively weak experimental support [97].

In randomly perturbed LCs on commonly observes a suppressed value of the paranematic-speronematic phase transition temperature $T_{I\;N}^{\left(p\right)}$ with respect to a bulk LC sample for a weak enough disorder. There are several papers in which the $T_{I\;N}^{\left(p\right)}=T_{I\;N}^{\left(p\right)}\;(R)$ dependence is analysed in detail [32, 71, 95]. It has been shown that in addition to randomness also surface ordering and in some cases even finite size effect should be taken into account [95]. In particular surface local ordering gives rise to enhanced LC ordering at the LC–perturber interface the thickness of which equals few *nm*. Such ordering could be in some cases well reproduced using a relatively simple bicomponent model [71], as demonstrated in the $T_{I\;N}^{\left(p\right)}\;(R)$ analysis in CPG and aerosil samples. In this model the average LC ordering is described by two order parameters, describing the average degree of ordering within the LC–perturber interfaces and in the remaining LC body.

Furthermore, for small enough values of *R*, *i.e.,* for a strong enough disorder, the 1st order speronematic-paranematic is replaced by gradual evolution of nematic ordering on varying temperature. The corresponding critical value of *R ≡ R_c_* is in general comparable to the nematic correlation lengh at *T_IN_*. This effect has been observed in LC-aerosil [24], LC-aerogel [27] and LC-CPG samples [96]. More detail analyses of RAN-type models yield approximate reationship between disorder anchoring strength and material-geometrical characteristics of samples. Furthermore, numerical estimates give reasonable quantitative estimates on critical values of *R_c_* [71, 96].

Note that the transition from the critical to the noncritical LC temperature behavior on decreasing *R* could be also triggered by an ordered surface due to noncritical character of the LC-interface coupling [98]. However, in such case $T_{I\;N}^{\left(p\right)}\;(R)$ would monotonously increase with decreasing *R* in the regime *R < R_c_* what is not observed.

## 5. Concluding Remarks

We have presented typical phase and structural behavior of randomly perturbed systems exhibiting continuous symmetry breaking using nematic liquid crystal phase as a testing ground. In liquid crystalline materials several features can be experimentally probed due to relatively good experimental accessibility, which can be exposed to different strengths and types of disorder. The impact of disorder on liquid crystal ordering is pronounced due to the softness of liquid crystal phases.

We have reviewed behavior of a nematic liquid crystal experiencing quenched random anisotropy field disorder assuming domain-type orientational ordering. Typical ordering of such systems has been demonstrated using relatively simple numerical simulations based on Lebwohl–Lasher lattice type model. It has been shown that a domain-type pattern characterized by a single characteristic length in the symmetry breaking field is inevitably formed at least temporally in the lower symmetry state (nematic) after a quench from the higher symmetry phase (isotropic) even in the absence of disorder. Such domain pattern can be stabilized by impurities. If impurities impose a random field-type disorder a domain pattern again characterized by a single characteristic length obeying the Imry–Ma scaling can be formed. We have presented cases in which the Imry–Ma scaling is expected and have shown that memory effects could be pronounced.

Assuming the domain-type nematic structure, we have used the Random Anisotropy Nematic (RAN) phenomenological model to study the phase behavior of nematic (speronematic)-isotropic (paranematic) phase transition. It has been shown that the main features which such a simple approach predicts are indeed realized in several experimental systems, *i.e.,* liquid crystal immersed in Controlled Pore Glasses, liquid crystal–aerosil dispersions, and other liquid crystal–impurities mixtures. The review indicates that such relatively simple RAN-type approaches are useful to predict qualitative behavior and make quantitative estimates on several physical properties of randomly perturbed systems, the phases of which are reached via a continuous symmetry breaking phase transition.
